# Altered functional connectivity and regional brain activity in a triple-network model in minimally conscious state and vegetative-state/unresponsive wakefulness syndrome patients: A resting-state functional magnetic resonance imaging study

**DOI:** 10.3389/fnbeh.2022.1001519

**Published:** 2022-10-10

**Authors:** Yituo Wang, Shanshan Chen, Xiaoyu Xia, Ying Peng, Bing Wu

**Affiliations:** ^1^Department of Radiology, Seventh Medical Center of Chinese PLA General Hospital, Beijing, China; ^2^Beijing Engineering Research Center of Mixed Reality and Advanced Display, School of Optics and Photonics, Beijing Institute of Technology, Beijing, China; ^3^Senior Department of Neurosurgery, First Medical Center of Chinese PLA General Hospital, Beijing, China; ^4^Department of Neurosurgery, Hainan Hospital of Chinese PLA General Hospital, Sanya, China; ^5^The Second School of Clinical Medicine, Southern Medical University, Guangzhou, China

**Keywords:** vegetative state/unresponsive wakefulness syndrome (VS/UWS), minimally conscious state (MCS), resting-state fMRI, functional connectivity, fractional amplitude of low-frequency fluctuation (fALFF), triple-network model

## Abstract

The purpose of this study was to investigate changes in functional connectivity and regional brain activity between and within the default mode network (DMN), salience network (SN), and executive control network (ECN) among individuals with disorders of consciousness (DOC) in the conditions of minimally conscious state (MCS) and vegetative-state/unresponsive wakefulness syndrome (VS/UWS). Twenty-five VS/UWS patients, 14 MCS patients, and 30 healthy individuals as normal control, completed resting-state fMRI scans. ROI-wise functional connectivity and fractional amplitude of low-frequency fluctuation (fALFF) were implemented to examine group differences. All ROI-wise and fALFF analyses masks were identified from the triple-network model. ROI-wise analyses indicated significantly decreased functional connectivity between posterior cingulate cortex (DMN)-left anterior insula (SN), right anterior insula (SN)-left dorsolateral prefrontal cortex (ECN), and right anterior insula (SN)-right amygdala (SN) in VS/UWS patients compared to MCS patients. Moreover, fALFF were observed reduced in the triple-network across all DOC patients, and as the clinical manifestations of DOC deteriorated from MCS to VS/UWS, fALFF in dorsal DMN, anterior/posterior SN, and left ECN became significantly reduced. Moreover, a positive correlation between fALFF of the left ECN and Coma Recovery Scale-Revised (CRS-R) total scores was found across all DOC patients. These findings contribute to a better understanding of the underlying neural mechanism of functional connectivity and regional brain activity in DOC patients, and this triple-network model provides new connectivity pattern changes that may be integrated in future diagnostic tools based on the neural signatures of conscious states.

## Introduction

Understanding the neural mechanisms underlying disorders of consciousness (DOC) remains one of the challenging issues in neuroscience. Clinically, DOC patients were mainly diagnosed with two specific conditions, including minimally conscious state (MCS) or vegetative-state/unresponsive wakefulness syndrome (VS/UWS) (Giacino et al., 2014). The former shows reproducible but inconsistent conscious behaviors (Giacino et al., 2002) while the latter represents a lack of identifiable communication or purposeful behavioral reactions to external stimuli (Laureys et al., 2010). It is usually difficult to exactly discriminate from neuroimaging metrics between MCS and VS/UWS patients, although there is a clear definition in clinical symptoms to distinguish between the two conditions (Giacino et al., 2014; Noirhomme et al., 2017).

In recent years, a number of resting-state functional magnetic resonance imaging (rs-fMRI) studies have demonstrated that MCS and VS/UWS patients are related to atypical patterns of functional connectivity or abnormal regional brain activity in large-scale brain networks (Vanhaudenhuyse et al., 2010; Soddu et al., 2012; Qin et al., 2015; Wu et al., 2015). Of the numerous brain functional networks, a triple-network model, consisting of three core intrinsic networks for human brain activity, consisting of the default mode network (DMN), the salience network (SN), and the executive control network (ECN). The DMN is thought to be involved in some aspects of internal or self-referential thoughts, with key nodes in the posterior cingulate cortex (PCC) and medial prefrontal cortex (mPFC) (Davey et al., 2016). The SN, plays crucial roles in the processes of information detection and integration, with key nodes in the dorsal anterior cingulate cortex (dACC) and anterior insula (Menon and Uddin, 2010). The ECN, containing widespread fronto-parietal cortices, displays strong activation during multifarious cognitive tasks (Sridharan et al., 2008). Our previous study (Chen et al., 2018) showed a decreased functional connectivity between the pontine tegmentum and the extensive cortical areas involving the ECN in patients with DOC. In addition, rs-fMRI regional homogeneity within the DMN-SN-ECN network model was observed reduced in both MCS and VS/UWS patients (Wang et al., 2022). However, the intrinsic functional connectivity and regional brain activity differences in this triple-network model between MCS and VS/UWS remains incompletely understood.

In this study, we aimed to investigate the specific changes of both between-network and within-network functional connectivity, and that of fractional amplitude of low-frequency fluctuation (fALFF) within the triple-network model using rs-fMRI in three groups of participants, which consist of MCS patients, VS/UWS patients, and healthy normal controls. We hypothesized that (a) because of the switching role of salience processing in the DMN and the ECN (Uddin, 2015), between-network functional connectivity in SN-DMN and SN-ECN and/or within-network functional connectivity in SN would be more disrupted in VS/UWS patients relative to MCS patients. (b) fALFF within the triple-network model, including the dorsal/ventral DMN, the anterior/posterior SN, and the left/right ECN, would demonstrate different levels of reduction as the clinical manifestations of DOC deteriorated from MCS to VS/UWS patients. Moreover, (c) a DOC-dependent alteration of functional connectivity and/or fALFF within the triple-network model might correlate with consciousness assessment using the Coma Recovery Scale-Revised (CRS-R).

## Materials and methods

### Participants

A total of 39 DOC patients were enrolled in the present study, with 14 MCS patients (male 8) and 25 VS/UWS patients (male 12) at Seventh Medical Center of Chinese PLA General Hospital from 2013 to 2016, which were the same population in our previous study (Wang et al., 2022). Thirty age-matched healthy individuals (male 15) were recruited as normal controls (NC). Table 1 outlined the clinical features of the MCS and VS/UWS patients which were recruited. The exclusion criteria were as follows: history of drug and/or alcohol abuse, history of mental diseases, or any contraindications for MRI scanning.

**TABLE 1:** Demographic and clinical features of DOC patients.

Patients ID	Gender	Age	Etiology	Lesion information	Months since onset	CRS-R total score	Diagnosis
1	M	39	ICH	Brainstem hemorrhage	less-than 1	17	MCS
2	F	27	TBI	Multi-focal contusion and left frontoparietal hemorrhage	10	12	MCS
3	M	23	TBI	Diffused axonal injury and subcortical atrophy	6	9	MCS
4	M	46	ICH	Brainstem hemorrhage	2	6	MCS
5	M	61	ICH	Left basal ganglia and thalamus hemorrhage	2	11	MCS
6	M	42	ICH	Left thalamus hemorrhage	3	7	MCS
7	M	53	ICH	Brainstem hemorrhage	7	11	MCS
8	F	33	TBI	Right frontoparietal lesion	12	7	MCS
9	M	23	TBI	Right basal ganglia lesion	10	7	MCS
10	F	67	ICH	Left thalamus hemorrhage	9	6	MCS
11	M	39	TBI	Brainstem atrophy	5	7	MCS
12	F	47	ICH	Right basal ganglia hemorrhage	47	8	MCS
13	F	31	TBI	Left thalamus and brainstem atrophy	3	11	MCS
14	F	68	TBI	Bilateral frontal lesion	less-than 1	7	MCS
15	F	64	ICH	Left thalamus hemorrhage	1	8	VS/UWS
16	F	42	AFE	n/a	less-than 1	7	VS/UWS
17	F	26	ICH	Right temporal lobe hemorrhage	4	6	VS/UWS
18	M	51	CA	Multi-focal cerebral infarction involving basal ganglia	3	8	VS/UWS
19	F	35	CA	Bilateral frontoparietal lesion	2	7	VS/UWS
20	F	38	AFE	n/a	2	6	VS/UWS
21	F	58	TBI	Left parietal-occipital lesion and brainstem hemorrhage	2	3	VS/UWS
22	M	23	TBI	Bilateral frontoparietal lesion	5	7	VS/UWS
23	M	36	HIE	n/a	1	7	VS/UWS
24	M	67	ICH	Right basal ganglia hemorrhage	1	5	VS/UWS
25	M	52	ICH	Brainstem hemorrhage	4	5	VS/UWS
26	M	39	ICH	Left basal ganglia hemorrhage	3	7	VS/UWS
27	M	41	CA	Multi-focal cerebral infarction involving basal ganglia	2	5	VS/UWS
28	F	53	ICH	Brainstem hemorrhage	3	5	VS/UWS
29	M	45	ICH	Left basal ganglia hemorrhage	2	9	VS/UWS
30	F	35	HIE	Anoxia caused by anesthesia	3	6	VS/UWS
31	M	45	CI	Left middle cerebral artery territory	less-than 1	7	VS/UWS
32	M	46	TBI	Multi-focal contusion	4	5	VS/UWS
33	F	40	CI	Left anterior and middle cerebral artery territory	2	7	VS/UWS
34	M	34	TBI	Diffused axonal injury and Brainstem atrophy	1	6	VS/UWS
35	F	28	TBI	Left frontoparietal lesion	1	11	VS/UWS
36	F	71	TBI	Diffused axonal injury and right tempo-parietal lesion	less-than 1	4	VS/UWS
37	M	45	TBI	Bilateral frontoparietal lesion	1	6	VS/UWS
38	F	69	ICH	Right thalamus hemorrhage	4	6	VS/UWS
39	F	35	HIE	n/a	3	7	VS/UWS

DOC, disorders of consciousness; F, female; M, male; MCS, minimally conscious state; VS/UWS, vegetative-state/unresponsive wakefulness syndrome; ICH, Intracranial hemorrhage; TBI, Traumatic brain injury; AFE, Amniotic Fluid Embolism; CA, Cardiopulmonary arrest; HIE, Hypoxic-ischemic encephalopathy; CI, Cerebral infarction.

On the day of rs-fMRI scanning, clinical consciousness assessments for each patient were evaluated using the CRS-R by clinical neuropsychologists with specialized training. The CRS-R, consisting of six subscales examining visual, auditory, motor, verbal, communication, and arousal functions, is recognized as a sensitive tool for detecting low-level consciousness and distinguishing between MCS and VS/UWS patients.

The local Ethics Committee of Seventh Medical Center of Chinese PLA General Hospital authorized the present study. Written informed consents from the NC group and each patient’s legal surrogate were obtained.

### Imaging data acquisition

Rs-fMRI and structural images were acquired using a Signa 3T MRI scanner (GE Healthcare, USA) with a standard head coil. A total of 210 functional images were acquired during rest. The parameters of the gradient echo-planar imaging sequence were shown as follows: repetition time (TR) 2000 ms, echo time (TE) 25 ms, slice order Interleave, slice thickness 4.0 mm, number of slices 39, flip angle 90°, matrix 64 × 64, field of view (FOV) 240 mm. A total of 176 high-resolution structural images were acquired before rs-fMRI scans. The parameters of the 3D T1-weighted spoiled gradient recalled echo sequence were shown as follows: TR 8.2 ms, TE 3.2 ms, slice thickness 1.0 mm, number of slices 188, flip angle 7°, matrix 256 × 256, FOV 256 mm.

### Imaging data analysis

Pre-processing were using Data Processing Assistant for Resting-State fMRI (DPARSF),^1^ a Matlab (R2016b) toolbox, which is based on statistical parametric mapping software (SPM8, UCL, London) and REST software.^2^ To prevent transitory signal fluctuations before magnetization achieving a steady-state, the first 10 time-points were removed. For the remaining images, slice-timing correction was performed. A Friston 24-parameter model was used for motion correction, and subsequently co-registered into the structural images. All functional images were then normalized using the Montreal Neurological Institute template (MNI152). Next, the rs-fMRI signals were regressed with a set of regressors as follows: the 24 motion parameters, the signal averaged over the white matter mask, cerebrospinal fluid mask and global mask. Subsequent data pre-processing included linear detrend and spatial smoothing by using a 6 mm full-width half-maximum (FWHM) Gaussian kernel. Calculating fALFF required the total value of the full-band amplitude, therefore bandpass filtering within 0.01–0.1 Hz was only used for further ROI-wise functional connectivity analysis.

Two analysis methods were applied to investigate functional connectivity and regional brain activity differences in MCS, VS/UWS, and NC groups. The first analysis was regions of interest (ROI)-wise where 16 ROI masks were chosen to cover the triple-network model described in our hypotheses (see Supplementary Figures 1–3 of Supplementary material). ROIs were defined using spheres 9 mm in radius or selected according to the published atlas of functional Regions of Interest (fROIs) from the Stanford Functional Imaging in Neuropsychiatric Disorders (FIND) Lab (Shirer et al., 2012). For the DMN, the selected masks were the posterior cingulate cortex (PCC) (centered around MNI co-ordinates –6, –52, 40), the medial PFC (mPFC) (–1, 45, 5), the subgenual ACC (sgACC) (0, 24, –8), and the left and right precuneus (fROIs). For the SN, the selected masks were the dorsal anterior cingulate cortex (dACC) (0, 24, 38), the left and right anterior insula (LAI and RAI) (fROIs) and the left and right amygdala (LAMY and RAMY) (fROIs). For the ECN, the selected masks were the left dorsolateral prefrontal cortex (LDLPFC) (–36, 15, 57), the right DLPFC (RDLPFC) (51, 15, 48), the left anterior inferior parietal lobule (aIPL) (–38, –52, 40), the right aIPL (38, –50, 42), the left superior parietal lobule (SPL) (–28, –60, 44), and the right SPL (32, –60, 42). The ROI-wise analysis used a 16 × 16 matrix of all feasible connections between all ROIs to assess functional connectivity differences across groups. The significance tests were using GRETNA software.^3^ The false discovery rate (FDR) approach with a corrected threshold of *p* < 0.05 (ROI-level) was used to account for multiple comparisons.

The second analysis was the fALFF measurement. First, fast Fourier transformation of whole-brain signal time series was to generate a frequency domain power spectrum. Since the power of a particular frequency was proportionate to the square of the amplitude of this frequency component, the square root was calculated by the power spectrum and averaged across 0.01–0.1 Hz at each voxel. The total ALFF value was obtained by summing the ALFF values in this range, and the fALFF value was calculated by dividing the total value of the full-band amplitude from 0.01 to 0.25 Hz. Meanwhile, the fALFF value was normalized to the Z-value (zfALFF) in order to reduce individual variances in overall whole-brain fALFF values. The masks of the three neural networks were first obtained from the fROIs (Shirer et al., 2012), including dorsal and ventral DMN, anterior and posterior SN, and left and right ECN. Subsequently, the fALFF values were extracted from the six masks and statistically analyzed by one-way ANOVA with LSD *post hoc* tests (α = 0.05) in MCS, VS/UWS, and NC groups.

### Correlation with the CRS-R total scores

All the extracted Fisher’s *Z*-values for significant changes in the ROI-wise and the fALFF analyses in the triple-network model were analyzed in SPSS Version 22 (IBM Corp., Armonk, NY, USA). Correlation analyses were using non-parametric tests (Spearman rank correlations). As these were exploratory secondary analyses an uncorrected *p* < 0.05 value was used.

## Results

### Demographic results

Demographic and clinical features of the enrolled DOC patients have been showed in Table 1.

#### Resting state ROI-wise functional connectivity differences within the triple-network model between minimally conscious state and vegetative-state/unresponsive wakefulness syndrome patients

**FIGURE 1:**
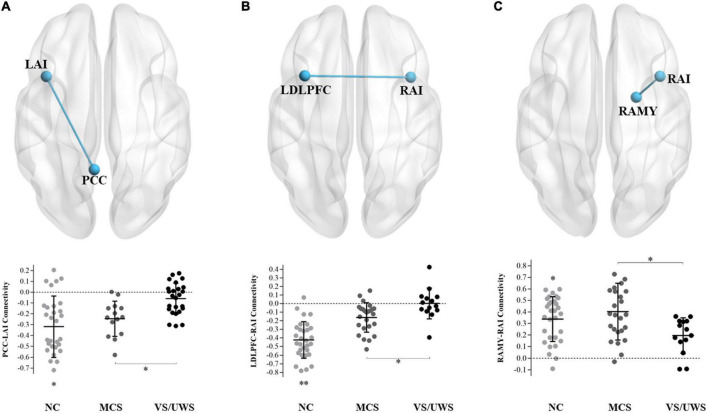
Connectivity differences between MCS and VS/UWS patient groups and NC group in ROI-wise analyses. Between-group comparisons of the ROI-wise analyses indicated significantly decreased functional connectivity between PCC-LAI **(A)**, RAI-LDLPFC **(B)**, and RAI-RAMY **(C)** during rest, in the VS/UWS group relative to MCS and NC groups. The graphs illustrate the differences in functional connectivity of PCC-LAI, RAI-LDLPFC, and RAI-RAMY in MCS, VS/UWS patients and NC groups (AI, anterior insula; PCC, posterior cingulate cortex; DLPFC, dorsolateral prefrontal cortex; AMY, amygdala; L, left; R, right; NC, healthy control individuals; MCS, minimally conscious state; VS/UWS, vegetative-state/unresponsive wakefulness syndrome; *p < 0.05; ^**^p < 0.01; error bars represent the standard error of measurement).

Figure 1 illustrated that the VS/UWS group was found to exhibit a significant level of decreased functional connectivity between PCC and LAI [*t*_(66)_ = –3.68, FDR *p* = 0.039], between RAI and LDLPFC [*t*_(66)_ = –2.91, FDR *p* = 0.049], and between RAI and RAMY [*t*_(66)_ = –3.32, FDR *p* = 0.043] compared with the MCS group, after adjusting for multiple comparisons. The VS/UWS group also showed lower connectivity than NC group, but the MCS group showed lower connectivity than NC group between PCC-LAI and between RAI-LDLPFC. There were no functional connectivity differences between RAI and RAMY between MCS and NC group.

#### Resting state fALFF differences within the triple-network model between minimally conscious state and vegetative-state/unresponsive wakefulness syndrome patients

**FIGURE 2:**
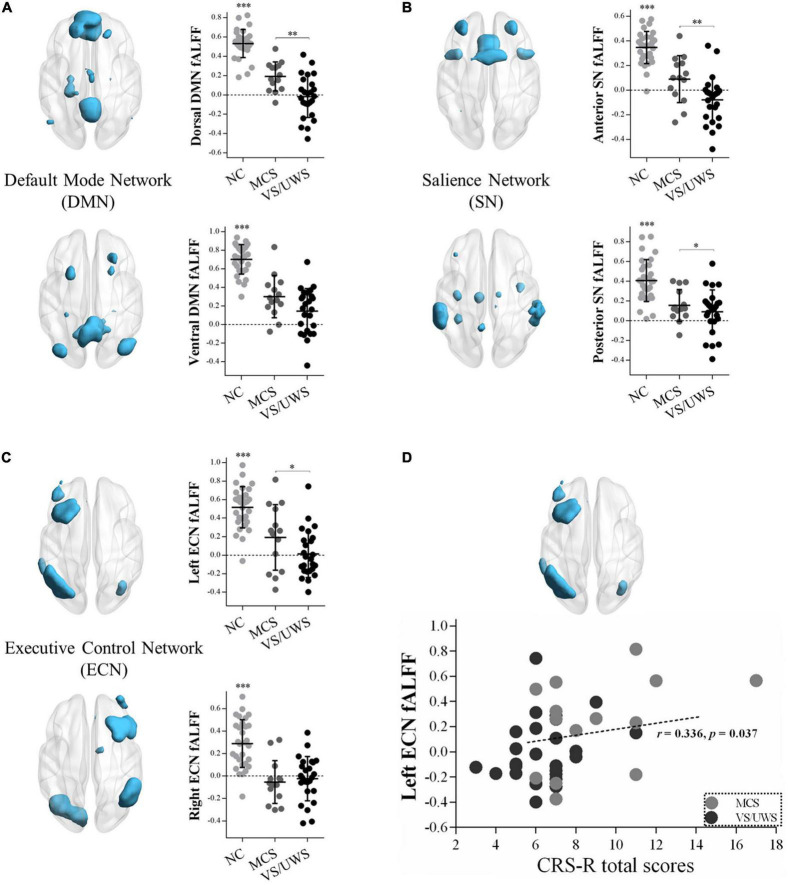
fALFF differences within the triple-network model and correlations with CRS-R total scores in MCS and VS/UWS patient groups and NC group. Reduced fALFF within the masks of dorsal/ventral DMN **(A)**, anterior/posterior SN **(B)**, and left/right ECN **(C)** were shown. As the clinical manifestations of DOC deteriorated from MCS to VS/UWS, fALFF within dorsal DMN (**A** top), anterior/posterior SN (**B** top/bottom), and left ECN (**C** top) became significantly reduced. Correlations between fALFF of the left ECN and CRS-R total scores were found across all DOC patients **(D)** (fALFF, fractional amplitude of low-frequency fluctuation; CRS-R, Coma Recovery Scale-Revised; DOC, disorders of consciousness; NC, healthy control individuals; MCS, minimally conscious state; VS/UWS, vegetative-state/unresponsive wakefulness syndrome; *p < 0.05; ^**^p < 0.01; ^***^p < 0.001; error bars represent the standard error of measurement).

Figure 2 illustrated significant differences of the extracted fALFF values in masks of the triple-network model. One-way ANOVA revealed significant main effects of the dorsal DMN [*F*_(2,66)_ = 45.92, *p* < 0.001], the ventral DMN [*F*_(2,66)_ = 17.46, *p* < 0.001], the anterior SN [*F*_(2,66)_ = 68.39, *p* < 0.001], the posterior SN [*F*_(2,66)_ = 52.21, *p* < 0.001], the left ECN [*F*_(2,66)_ = 25.43, *p* < 0.001], and the right ECN [*F*_(2,66)_ = 21.58, *p* < 0.001] in the three groups. Subsequently, LSD *post hoc* tests showed that the VS/UWS group exhibited a significant level of decreased fALFF in the dorsal DMN [LSD-t_(66)_ = –3.05, *p* = 0.003], the anterior SN [LSD-t_(66)_ = –3.57, *p* = 0.001], the posterior SN [LSD-t_(66)_ = –2.26, *p* = 0.027], and the left ECN [LSD-t_(66)_ = –2.02, *p* = 0.047] during rest, relative to MCS group. There were no fALFF differences of the ventral DMN and the right ECN between MCS and VS/UWS group.

#### Correlations between neural differences and CRS-R total scores in minimally conscious state and vegetative-state/unresponsive wakefulness syndrome patients

Figure 2 illustrated a positive correlation between fALFF values of the left ECN and CRS-R total scores across all DOC patients (*r* = 0.336, *p* = 0.037), while in the MCS or VS/UWS group separately, the linear trend was not found. There were no significant correlations between PCC-LAI, RAI-LDLPFC, and RAI-RAMY functional connectivity and CRS-R total scores across both MCS and VS/UWS patients. Similarly, there were no significant correlations between fALFF values of the dorsal and ventral DMN, anterior and posterior SN, and right ECN and CRS-R total scores across both MCS and VS/UWS patients.

## Discussion

In this study, on the basis of the triple-network model, we observed the functional dysconnectivity between and within the DMN, SN, and ECN in MCS and VS/UWS patients. VS/UWS group showed decreased between-network connectivity between the PCC (DMN)-LAI (SN) and the RAI (SN)-LDLPFC (ECN), and also demonstrated decreased within-network connectivity between the RAI (SN) and RAMY (SN), compared with MCS group. Moreover, reductions of fALFF values were observed in the triple-network across all DOC patients compared with NC group, and as the clinical manifestations of DOC deteriorated from MCS to VS/UWS patients, fALFF in dorsal DMN, anterior/posterior SN, and left ECN showed great reductions. Notably, a positive correlation between fALFF of the left ECN and CRS-R total scores was observed in all DOC patients, however the linear trend was not found in MCS or VS/UWS patients, respectively. These findings contribute to a better understanding of the underlying neural mechanism of functional connectivity and regional brain activity in DOC patients, and this triple-network model provides new connectivity pattern changes that may be integrated in future diagnostic tools based on the neural signatures of conscious states.

In our previous study, at the whole-brain level, we found that MCS and VS/UWS patients demonstrated DOC-dependent reduced regional homogeneity in the posterior cingulate cortices, medial prefrontal cortices, and bilateral fronto-parieto-temporal cortices, which were also the core brain areas of the triple-network model (Wang et al., 2022). In order to further investigate the changes in functional connectivity between core hubs in the triple-network model, the ROI-wise functional connectivity analyses were performed. Of the triple-network model, the DMN, which were known to be involved in self-consciousness and mind-wandering, has been extensively investigated in DOC patients (Cruse et al., 2011; Stender et al., 2014; Monti et al., 2015; Laforge et al., 2020). These studies highlighted that the degree of activity and connectivity of the DMN might help differentiate patients with MCS and VS/UWS (Porcaro et al., 2021). As one of the key nodes of the DMN, a functional disconnection of the PCC within the DMN (Soddu et al., 2012) and between other intrinsic networks (Silva et al., 2015) in DOC patients. Wu et al. (2015) found decreased functional connectivity strength with loss of consciousness between PCC and AI, which is one of the key nodes of the SN. Another study has shown that PCC was hypoconnected to insula between VS/UWS and MCS patients (Di Perri et al., 2016). These observations supported our finding of dysconnectivity between the DMN and the SN in VS/UWS patients compared with MCS patients. The SN plays crucial roles in monitoring behavior-dependent salient stimulus and mediating dynamic switching between the DMN and the ECN to guide appropriate responses to salient stimulus (Uddin, 2015). In our study, we found the abnormal connection between the DMN and the SN, which might be indicating evidence of disrupted cortical information integration.

Another major finding of our study is decreased functional connectivity between the RAI (SN)-LDLPFC (ECN) and the RAI (SN)-RAMY (SN) in the VS/UWS group, compared with the MCS group. The RAI node of the SN is assumed to be involved in brain network dynamics coordination (Menon and Uddin, 2010). In a task-based fMRI study, activations in the insula in the VS/UWS were shown to be lower than those in the MCS during the presentation of emotionally salient sounds (Kotchoubey et al., 2013). Emerging evidence indicated that the RAI generated control signals that causally influenced the ECN and DMN (Uddin et al., 2011; Supekar and Menon, 2012). These results may reflect that functions which depend on an intact salience network including high-level cognitive control and self-oriented cognition seem to be compromised to some extent in MCS and VS/UWS patients. Notably, the RAI can be subdivided into the dorsal and ventral portion (Chang et al., 2013). The former is associated with higher-level cognitive processes (such as task switching), whereas the latter is related with emotional processes (such as emotions perception). The VS/UWS group in this study demonstrated more decreased functional connectivity between the RAI and right amygdala, a subcortical region involved in emotion processing which is also a component of the SN (Hermans et al., 2014), demonstrating a decrease in the integration of external sensory information with internal emotional and body-state signals, consistent with a prior finding (Qin et al., 2015). However, we have not found statistically significant differences of within-SN functional connectivity between the MCS and NC group.

The fALFF, which is determined by measuring the square root of the power spectrum between 0.01 and 0.1 Hz, provides an indication of oscillation strength for the integrity of brain networks (Yang et al., 2007). The DOC patients showed reduced fALFF in the triple-network model, compared with the NC group. Remarkably, as the clinical manifestations of DOC deteriorated from MCS to VS/UWS, the fALFF of dorsal DMN, anterior/posterior SN, and left ECN became significantly reduced. This is consistent with our previous study using regional homogeneity analyses (Wang et al., 2022), which provides mutual support for the results of both studies. Based on spatial cues and novelty or salience cues, the DMN can be subdivided into the dorsal and ventral portion (Corbetta and Shulman, 2002; Hannawi et al., 2015). The former is more involved in top-down attention through targeted spatial exploration, and the latter is associated with bottom-up attention by purposeless space detection. Thus, our findings suggest that top-down attention may be associated with the state of awareness, whereas the impaired bottom-up attention seems to be a prevalent feature in patients with DOC. The areas within anterior SN overlap most of the conventional SN, while the posterior SN contains partial areas of dorsal DMN including the PCC and precuneus (Sridharan et al., 2008; Cazzoli and Chechlacz, 2017). So there is reason to believe that fALFF of both anterior and posterior SN displayed varying reduction according to the level of consciousness, as we mentioned above. In addition, a positive correlation between fALFF of the left ECN and CRS-R total scores was observed across all DOC patients. So far, we have not seen a statistically significant linear trend in either the MCS or the VS/UWS groups. Hence the stability and reliability are needed to be more clearly understood in future works.

There are various limitations to the present study. First, it is challenging to accurately describe whether the observed functional dysconnectivity and reduced fALFF within the triple-network model are the cause or result of the behaviorally observed dissociation of awakening-consciousness in patients with DOC. Therefore, other analytical methodologies, such as Granger causality analysis, might be used to evaluate this issue. Second, based on an analysis that the insula could be subdivided into three portions (Chang et al., 2013), further seed-based analysis according to the distinct nuclei is required. Third, the positive correlation between fALFF values of the left ECN and CRS-R total scores across all DOC patients might be due to the number of subjects included in the correlation analysis, i.e., when we included more subjects in the analysis, we might get the significant difference. Future studies will include more subjects to confirm these results. Fourth, it was unfortunate that the number of CRS-R assessments was not provided in clinical data, as it was demonstrated that at least 5 CRS-Rs are preferable to reduce the risk of misdiagnoses (Wannez et al., 2017). Fifth, there were two MCS and three VS/UWS patients that underwent rs-fMRI scans less than 1 month after acute brain damage. Although the current diagnoses were in line with the Royal Collection of Physicians guidelines, which recommend a vegetative state should be persistent 1 month after acute brain damages, a thorough consolidation of clinical symptoms among these patients may not have been done, the influence on the study should however remain modest.

## Conclusion

In conclusion, we found that the VS/UWS group showed decreased functional connectivity between the DMN-SN and between the SN-ECN, and within the SN, compared with the MCS group. Moreover, fALFF were observed reduced in the triple-network across all DOC patients compared with the NC group, and as the clinical manifestations of DOC deteriorated from MCS to VS/UWS, fALFF in dorsal DMN, anterior/posterior SN, and left ECN became significantly reduced. In addition, a positive correlation between fALFF of the left ECN and CRS-R total scores was found across all DOC patients. These findings contribute to a better understanding of the underlying neural mechanism of functional connectivity and regional brain activity in DOC patients, and this triple-network model provides new connectivity pattern changes that may be integrated in future diagnostic tools based on the neural signatures of conscious states.

## Data availability statement

The raw data supporting the conclusions of this article will be made available by the authors, without undue reservation.

## Ethics statement

The studies involving human participants were reviewed and approved by the Local Ethics Committee of Seventh Medical Center of Chinese PLA General Hospital. The patients/participants provided their written informed consent to participate in this study.

## Author contributions

YW: study concept and design, analysis and interpretation of data, and original draft preparation. SC: study concept and design and acquisition of data. XX: analysis and interpretation of data and acquisition of data. YP: acquisition of data and analysis and interpretation of data. BW: study concept and design and critical revision of manuscript for intellectual content. All authors contributed to the article and approved the submitted version.

## Footnotes

1
http://rfmri.org/content/dparsf

2
http://restfmri.net/forum/restplus

3
https://www.nitrc.org/projects/gretna/
