# Supplementary material for: Altered functional connectivity and regional brain activity in a triple-network model in minimally conscious state and vegetative-state/unresponsive wakefulness syndrome patients: A resting-state functional magnetic resonance imaging study
Source: Front Behav Neurosci. 2022 Oct 10;16:1001519. doi: 10.3389/fnbeh.2022.1001519 (PMC9588962; doi:10.3389/fnbeh.2022.1001519)
Supplement: Supplementary file 1 [file Data_Sheet_1.PDF]

Supplementary Material

1 Selecting ROI seed regions for the triple-network model

To satisfy the criteria of our hypotheses, we selected ROIs from each of the triple-network model, the default mode network (DMN), the salience network (SN), and the executive control network (ECN).

We used research of (Williams,2016) to define the triple-network model and brain regions. *(Williams LM. 2016. Precision psychiatry: a neural circuit taxonomy for depression and anxiety. Lancet Psychiatry. 3(5): 472-80.)*

We used research of (Fox, et al.,2005) for the DMN (Fig. S1). *(Fox MD, Snyder AZ, Vincent JL, Corbetta M, Van Essen DC, Raichle ME. 2005. The human brain is intrinsically organized into dynamic, anticorrelated functional networks. Proc Natl Acad Sci U S A. 102(27): 9673-8.)*

We used research of (Menon, 2011) for the SN (Fig. S2). *(Menon V. 2011. Large-scale brain networks and psychopathology: a unifying triple network model. Trends Cogn Sci. 15(10): 483-506.)*

We used research of (Niendam et al., 2012) for the ECN (Fig. S3). *(Niendam TA, Laird AR, Ray KL, Dean YM, Glahn DC, Carter CS. 2012. Meta-analytic evidence for a superordinate cognitive control network subserving diverse executive functions. Cogn Affect Behav Neurosci. 12(2): 241-68.)*

2 Supplementary Figures

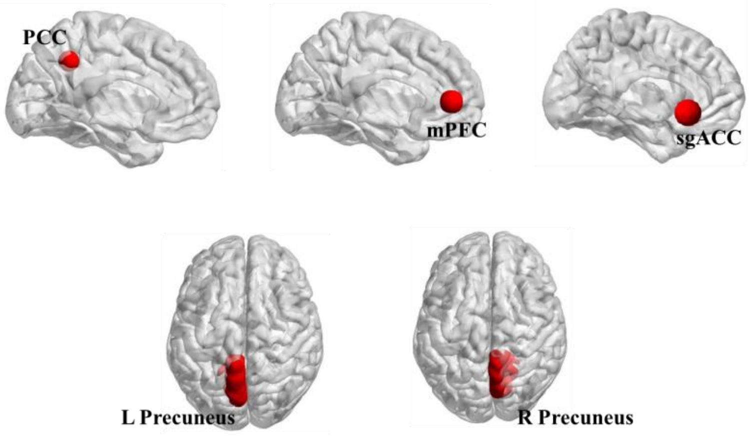

Fig. S1 The DMN used in the ROI-wise analysis and comprised of the posterior cingulate cortex (PCC), the medial PFC (mPFC), the subgenual ACC (sgACC), and the left and right precuneus.

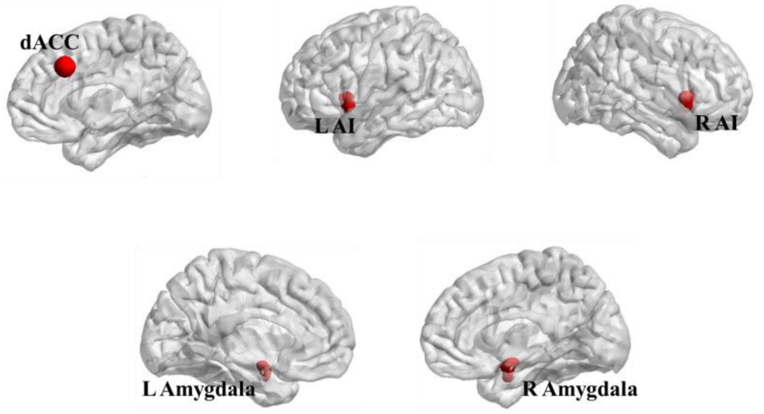

Fig. S2 The SN used in the ROI-wise analysis and comprised of the dorsal anterior cingulate cortex (dACC), the left and right anterior insula (LAI and RAI) and the left and right amygdala.

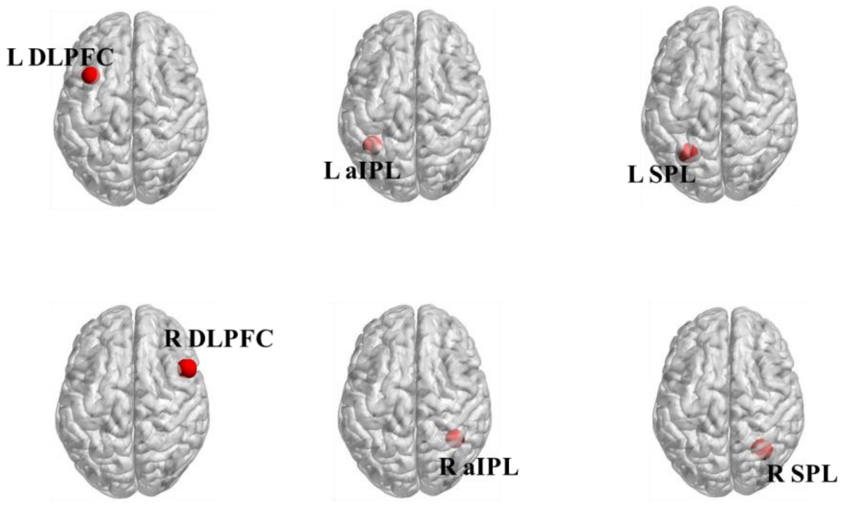

Fig. S3 The ECN used in the ROI-wise analysis and comprised of the left and right dorsolateral prefrontal cortex (LDLPFC and RDLPFC), the left and right anterior inferior parietal lobule (LaIPL and RaIPL), the left and right superior parietal lobule (LSPL and RSPL).
